# Osteopontin: an early innate immune marker of *Escherichia coli *mastitis harbors genetic polymorphisms with possible links with resistance to mastitis

**DOI:** 10.1186/1471-2164-10-444

**Published:** 2009-09-18

**Authors:** Karin Alain, Niel A Karrow, Catherine Thibault, Jessika St-Pierre, Martin Lessard, Nathalie Bissonnette

**Affiliations:** 1 Dairy and Swine Research and Development Centre, Agriculture and Agri-Food Canada, Sherbrooke, Quebec, J1M 1Z3, Canada; 2 Université de Sherbrooke, Sherbrooke, Quebec, J1H 5N4, Canada; 3 University of Guelph, Guelph, Ontario, N1G 2W1, Canada

## Abstract

**Background:**

Mastitis is the most important disease in dairy cows and it causes significant lost of profit to producers. Identification of the genes, and their variants, involved in innate immune responses is essential for the understanding of this inflammatory disease and to identify potential genetic markers for resistance to mastitis. The progeny of dairy cows would benefit from receiving favourable alleles that support greater resistance to infection, thus reducing antibiotic use. This study aims to identify a key gene in the innate immune response to mastitis, led us to evaluate its genetic association with somatic cell score (SCS), which is an indicator of clinical mastitis, and to evaluate its impact on other traits related to milk production.

**Results:**

The osteopontin transcript (*SPP1*) was identified in the somatic cells from cows experimentally infected with *Escherichia coli*. By selecting bulls with extreme estimated breeding values (EBVs) for SCS, which is an indicator of mammary gland health, four DNA polymorphisms in the *SPP1 *genomic sequence were found. Statistical analysis revealed that the SNP *SPP1c.-1301G>A *has an impact on EBV for SCS (*P *< 0.001) Using an allele substitution model, *SPP1c.-1251C>T*, *SPP1c.-430G>A*, and SPP1c.*40A>C have an impact on SCS whereas *SPP1c.-1301G>A *has an effect on the EBVs for milk yield (second and third lactations), fat and protein percentages (all three lactations). Analysis revealed statistically significant differences between haplotype groups at a comparison-wise level with sire EBVS for SCS for the first (*P *= 0.012), second (*P *< 0.001), and third (*P *< 0.001) lactations.

**Conclusion:**

This study reports the link between DNA polymorphisms of *SPP1*, the number of milk immune cells and, potentially, the susceptibility to mastitis. These SNPs were identified by *in silico *search to be located in transcription factor recognition sites which factors are presumably involved in the Th1 immune response and in the Th2 regulation pathway. Indeed, one SNP abolished the SP1 recognition site, whereas another SNP affected the transcription binding factor IKAROS. All together, these findings support the genetic potential of these variants in terms of selection for the improvement of mastitis resistance in dairy cows.

## Background

Mastitis is an inflammatory condition of the mammary gland caused primarily by microorganisms, usually bacteria, that invade the udder, multiply and secrete toxic products that are very harmful to the host. In Canada, environmental mastitis (clinical mastitis) is most commonly caused by *Escherichia coli*. This infection is generally short taking a few days to be eliminated by the immune system, but the animal presents severe clinical signs that include inflammation of the udder, milk clots and altered behaviour (fever, loss of appetite). With annual costs for the herd of approximately $180 per cow [1], mastitis is still the most commonly occurring disease in Canadian dairy herds. These important losses to producers result not only from early culling and treatment costs, but also from the adverse effects of the decrease in production, and the need to discard milk that is unfit for human consumption because it is infected or contains antibiotic residues [2,3].

The mammary gland is typically a sterile environment and, therefore, the entry of any foreign body usually triggers a localized immune response. The first line of defence against disease-causing microorganisms is the innate immune system, which induces mechanisms that are not pathogen species-specific [4]. Innate immune cells in the mammary gland are comprised of macrophages, granulocytes, natural killer cells, and dendritic and mammary epithelial cells [5]. These cells have receptors that recognize motifs or pathogen-associated molecular patterns (PAMP) on the surface of microorganisms. For example, the lipopolysaccharides on the surface of Gram-negative bacteria such as *E. coli *become attached to the phagocytic cells via Toll-like receptor 4 (TLR-4), whereas Toll-like receptor 2 (TLR-2) binds to Gram-positive motifs such as peptidoglycan or lipoteichoic acid on the surface of *S. aureus *[6].

Recognition of an invading pathogen activates cellular reactions, leading to the secretion of inflammatory mediators called cytokines. These signalling molecules trigger cellular communication, chemotaxis and lymphocyte differentiation. The cytokines include inflammatory interleukins-(IL)-1β, -6 and -12, tumour necrosis factor-alpha (TNF-α) and interferon-gamma (IFN-γ) [7]. Once phagocytic cells recognize and internalize pathogens, the cells present the pathogen's antigenic determinants to the T lymphocytes. Then these cells, in the presence of IL-12, differentiate into Th1 effector cells which are responsible for cell-mediated immunity. These Th1 cells produce inflammatory mediators such as IFN-γ, that enhance macrophage effectors functions against intracellular pathogens [8].

Macrophages are the predominant cells in the healthy mammary gland [9]. During intramammary infection, however, a release of inflammatory mediators, especially by macrophages, leads to the recruitment of neutrophils into infected quarters from the circulation. At this stage, these cells account for more than 90% of milk cells [10]. The neutrophils are responsible for the eventual elimination of the pathogens. For example, activated neutrophils degranulate and produce/secrete bactericidal components, namely reactive oxygen species (ROS) [11]. The recruitment of neutrophils into the mammary gland causes an increase in somatic cell count (SCC) that can reach more than 1,000,000 cells/mL during the course of an infection, whereas the SCC is normally less than 100,000 cells/mL in a healthy mammary gland [4].

Prevention and control of mastitis by improving the natural defence mechanisms is important not only for dairy producers but also for consumers, because of increased concerns about food safety, antibiotic use and animal welfare [12]. One approach would be to define breeding objectives with increased weight of health-related traits in genetic selection [13]. Genetic selection to increase antibody responsiveness seems to be possible, but the acquired immune response traits have proven to be inconsistent indicators of udder health [14]. Components of the adaptive immune system have been studied intensively, but there is still need for the development of efficient vaccine against pathogens that cause intramammary infection for bovine [12,15-17]. Unlike the adaptive immune system, some mechanisms of the innate immunity are conserved throughout the animal kingdom and can thus be thought of as general mechanisms responsible for broad environmental responses. These evolutionarily conserved systems have been analyzed in detail and include, among others, the complement gene family [18] and the Toll-like receptors (TLR) [19]. The parameters of innate immune responses can be used to study resistance to mastitis. These results can lead to the selection of breeding animals that carry favorable polymorphisms or alleles able to improve the resistance to infection of their offspring. [20].

Genetic parameters such as heritability and phenotypic and genetic correlations are useful statistical tools for measuring the genetic component of a trait or group of traits. These genetic parameters do not require any knowledge about the number of genes involved as they are estimated from correlations of phenotypic data among relatives. The present study is based essentially on the somatic cell score (SCS), because data for clinical mastitis prevalence are still not available for the Canadian dairy population [21]. However, the usefulness of SCS as an indirect selection tool for reducing mastitis has been reported in several studies [22-25]. Most estimates of the genetic correlation between SCS and clinical mastitis range from 0.50 to 0.80 [26-29]. In a recent study, the genetic correlation was observed to ranges from 0.55 to 0.93 [30]. These are reasonably high values which suggest that SCC and mastitis occurrence are partly caused by the expression of the same trait. The SCC distribution is often highly skewed and it is usually transformed on a logarithm scale, as follows: SCS = log_2 _(SCC/100,000) + 3 [22]. When used for genetic evaluation, bulls receive an estimated breeding value (EBV) based on the SCS records of their daughters. If dairy producers select bulls with a low EBV for SCS, this is expected to improve mastitis resistance in their herd [22,28,31]. The SCS has now become the most important indicator associated with the health of the mammary gland, but little is known about the key factors which regulate the number of somatic cells in milk from healthy cows. Thus, in the present study we investigated the early activated transcripts of immune cells of the lactating mammary gland in order to identify a key gene in the innate immune response to mastitis.

Osteopontin (or secreted phosphoprotein 1, *SPP1*) is a cytokine produced by macrophages and activated T cells [32]. Osteopontin has been described as an early component of the T cell activation mechanism. Enhanced in T cells, it recruits macrophages at the infection site and improves cell-mediated immunity (Th1) by inducing secretion of Th1 cytokines [33-36]. Osteopontin is a multi-faced protein [35,37]. It promotes bone remodelling [38], wound healing and survival of stressed cells [39], but is also associated with metastasis status and poor survival prognosis [40,41]. It was found associated with certain pathologies such as restenosis, formation of kidney stones, and autoimmune disease [37], but also found to confer resistance to several intracellular pathogen infections through recruitment and activation of macrophages [42]. In this study, we reported the detection of this abundant transcript, which is expressed early on during mastitis. Following the detection of this key gene in the mammary somatic cells isolated early during the immune response, we evaluated the association of *SPP1 *with SCC, which is the most widely used indicator of mastitis [31]. Identifying favourable *SPP1 *alleles for mastitis resistance would make it possible for dairy breeders to increase them in the Canadian Holstein population to potentially increase the natural resistance to mammary gland infection.

## Results

### Experimental mastitis and detection of the candidate gene

**Figure 1:**
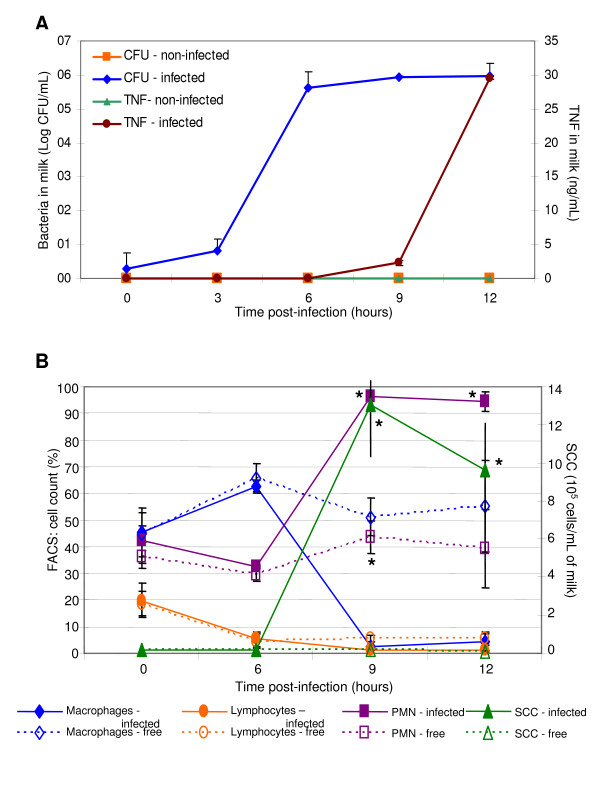
**Experimental mastitis**. Milk samples were analysed at different times for intramammary bacterial infection (pannel A; CFU, left y-axis), inflammatory mediator (pannel A; TNF-α; right y-axis), and for somatic cell profil (pannel B). Data are expressed as mean ± standard error of the mean. During the first 6 hours post-challenge, mean values of 4 cows are reported. By 6 hours, two cows were not sampled because they received antibiotics (Materials and Methods). Error bars, standard error of the means.

An infectious dose of 94 ± 8 CFU of a fresh exponential culture of *E. coli *was used and produced a bacterial infection in all challenged quarters (Figure 1A) of the four lactating cows: groups 6 h (n = 2) and 12 h (n = 2). The front control quarters, which had been infused with saline, remained free of bacteria. There were no apparent signs of clinical infection at 6 h whereas infection was confirmed by 12 h. These two cows showed signs of clinical infection: clotting in milk, fever (40°C), and local redness along with pain after 12 h. The tumour necrosis factor-alpha (TNF-α) was detectable in milk at 9 h post-infection (Figure 1A). Increases in both SCC and granulocytes in milk were highly correlated between 6 and 9 h post-challenge (Figure 1B), which confirmed that granulocytes were the major invading cells in the mammary gland upon infection. During the same 6-to-9-h period, macrophages drastically decreased in proportion (*P *< 0.05; Figure 1B). Only 2 to 4% remained detectable whereas in the bacteria-free quarters, a significant level (51 to 56%) of macrophages was maintained. The inflection point for this longitudinal survey was 6 h. Indeed, no changes in the distribution of lymphocytes, macrophages and granulocytes in milk, between infected quarters (continuous lines) and uninfected quarters (dashed lines) were apparent in the first 6 h post-infection (Figure 1B). We thus monitored the molecular events prior to the increase of granulocytes (inflection point) in milk. The differential transcripts of milk immune cells were analysed at 5 h post-infection. One hundred clones from the cDNA library of milk immune cells were sequenced (Material and Methods). The *SPP1 *transcript was identified using the Basic Local Alignment Search Tool (BLAST) to search against the National Center for Biotechnology Information (NCBI) database. This induction of *SPP1 *transcript was also observed in PBMC (peripheral blood mononuclear cells) challenged *in vitro *with heat-inactivated bacteria as described in Material and Methods. The increased *SPP1 *transcripts abundance in PBMC was detected within 6 h (up to 4 fold by 3 h) by real-time RT-PCR in three independent assays (data not shown).

### Analysis of the *SPP1 *genomic sequence

Search for DNA polymorphisms was performed by selecting bulls with extreme EBV for SCS (Material and Methods). Each different genomic region of *SPP1 *(promoter, 5' untranslated sequence [UTR], intron 1, exons 1-7 and 3' UTR) were sequenced as described in Materials and Methods. Comparison of the results of the electrophoregrams from the two groups presenting extreme EBV for the SCS trait uncovered differences in the allelic distribution of four nucleotides. Two single-nucleotide polymorphisms (SNPs) were found in the promoter. A novel SNP was identified and has been submitted to NCBI dbSNP on April 23^rd ^2009 (SNP submission number ss130452346). This novel transition SNP (*SPP1c.-1301G>A*) and the transition SNP *SPP1c.-1251C>T *were found respectively 1,301 and 1,251 nucleotides (nt) before the ATG (start) codon, which initiates translation from exon 2. The third transition SNP (*SPP1c.-430G>A*) was located in the first intron, 430 nt upstream from the start codon. Lastly, the transversion SNP (SPP1c.*40A>C) was found 40 nt downstream from the stop codon in exon 7 within the 3' UTR. No SNPs were found in the coding region. We scrutinized the electrophoregrams in the 2,428-to-2,419-nucleotide region upstream from the ATG gene corresponding to the insertion and deletion (INDEL) variation rs43702359 (NCBI dbSNP accession number) which had been reported elsewhere [43]. No such variation was observed in the population sequenced.

### SNPs and allelic frequencies

Genotypic and allelic frequencies of the four SNPs for the 578 bulls are summarized in Table 1. All SNPs were checked for conformance with the Hardy-Weinberg equilibrium, as determined by the Chi-square test. The frequency of the alleles varied in the population. Some alleles were present in low frequency, such as allele A (10.5%) for the SNP *SPP1c.-1301G>A*, which also explained the low abundance of homozygous bulls (1.0%) in the population tested. Homozygous bulls harbouring the genotype T for *SPP1c.-1251C>T *and genotype A for *SPP1c.-430G>A *were not frequent (6.4%), and the abundance of these alleles was 25.2% for both among the 578 genotyped bulls. These two SNPs were linked in the population tested: allele C for *SPP1c.-1251C>T *was linked to allele G for *SPP1c.-430G>A*, and *vice versa *(allele T linked to allele A). For the SNP SPP1c.*40A>C, 9.1% of the bulls were homozygous for allele C, a proportion of 30% in the population genotyped (Table 1).

**Table 1:** Genotype and allele frequencies of the SNPs detected in the bovine *SPP1 *gene.

**DNA polymorphisms**	**Genotype**	**Code**	**Frequency (%)**	**Allele**	**Frequency (%)**
*SPP1c.-1301G>A*	GG	-1	463 (80.1)	G	1035 (89.5)
	GA	0	109 (18.9)		
	AA	1	6 (1.0)	A	121 (10.5)
					
*SPP1c.-1251C>T*	CC	-1	324 (56.1)	C	865 (74.8)
	CT	0	217 (37.5)		
	TT	1	37 (6.4)	T	291 (25.2)
					
*SPP1c.-430G>A*	GG	-1	324 (56.1)	G	865 (74.8)
	GA	0	217 (37.5)		
	AA	1	37 (6.4)	A	291 (25.2)
					
SPP1c.*40A>C	AA	-1	287 (49.6)	A	813 (70.3)
	AC	0	239 (41.4)		
	CC	1	52 (9.1)	C	343 (29.7)

### SNP associations

The EBVs for SCS at the first, second and third lactations and for over all lactations are reported according to the genotypes for *SPP1c.-1301G>A*, *SPP1c.-1251C>T*, *SPP1c.-430G>A *and SPP1c.*40A>C (Table 2). Allele G of *SPP1c.-1301G>A *had a favourable impact on SCS EBV at any lactation (*P *< 0.001). The SNP *SPP1c.-1251C>T *and *SPP1c.-430G>A *did not have an effect on SCS EBV (global comparison - over all lactations), but genotypic comparison showed a significant difference for the third lactation only between homozygous and heterozygous bulls for the dominant allele (*P *= 0.046; data not shown). The statistical analysis also revealed a stronger association between SPP1c.*40A>C and EBVs for SCS as the number of lactations increased: first lactation (SCS1; *P *= 0.183), second lactation (SCS2; *P *= 0.014) and third lactation (SCS3; *P *= 0.004).

**Table 2:** Effects of polymorphisms in the bovine *SPP1 *gene on EBVs for SCS for the first, second, third, and over all lactations.

**SNP**	**Least squares mean^a^**			**(± SEM)^a^**	***P *value**
*SPP1c.-1301G>A*	GG	GA	AA		
SCS	2.98^b^	3.16^c^	3.38^c^	(± 0.14)	<.001
SCS1	2.98^b^	3.10^c^	3.25^b, c^	(± 0.13)	<.001
SCS2	2.96^b^	3.14^c^	3.39^c^	(± 0.15)	<.001
SCS3	2.99^b^	3.16^c^	3.50^c^	(± 0.16)	<.001
*SPP1c.-1251C>T*	CC	CT	TT		
SCS	3.04	2.97	3.02	(± 0.06)	0.067
SCS1	3.02	2.98	3.02	(± 0.05)	0.446
SCS2	3.03	2.96	3.00	(± 0.06)	0.155
SCS3	3.06^b^	2.97^c^	3.01^b, c^	(± 0.07)	0.057
*SPP1c.-430G>A*	GG	GA	AA		
SCS	3.04	2.97	3.02	(± 0.06)	0.067
SCS1	3.02	2.98	3.02	(± 0.05)	0.446
SCS2	3.03	2.96	3.00	(± 0.06)	0.155
SCS3	3.06^b^	2.97^c^	3.01^b, c^	(± 0.07)	0.057
SPP1c.*40A>C	AA	AC	CC		
SCS	3.06^b^	2.97^c^	2.98^b, c^	(± 0.05)	0.004
SCS1	3.03	2.98	2.98	(± 0.05)	0.183
SCS2	3.05^b^	2.95^c^	2.97^b, c^	(± 0.05)	0.014
SCS3	3.09^b^	2.97^c^	2.97^b, c^	(± 0.06)	0.004

^a ^The least square means are the adjusted means of the EBVs for the SCS for bulls which are grouped by genotype for each SNP and calculated for the respective lactation (over all, first, second or third lactations). SEM standard error of the mean.

^b, c ^Means (within a line) without a common superscript letter differ from each other at the 5% level of significance.

The estimated average allele substitution effects are presented in Table 3. No significant association of SNP *SPP1c.-1301G>A *was detected with SCS in any of the lactations. The SNP *SPP1c.-1251C>T *and *SPP1c.-430G>A *were significant at a comparison-wise level in association with EBVs for SCS (*P *= 0.014), SCS1 (*P *= 0.035), and SCS2 (*P *= 0.023). The estimated substitution effect was about the same for SCS3 but the larger SE crushed the statistical validity (*P *= 0.056). The allele substitution effects for *SPP1c.-1251C>T *for over all, first, and second lactations are respectively 0.117 ± 0.047, 0.097 ± 0.046 and 0.118 ± 0.052. Thus, the increase in SCS for allele T over allele C corresponded to 33% of the SD for SCS EBV. The same effects on EBV for SCS were found for *SPP1c.-430G>A*, but the negative allelic substitution effect carried by allele A had the opposite effect (reduced the EBV for SCS). Although it was not significant for the first lactation (SCS1; *P *= 0.121), the 3' UTR SNP SPP1c.*40A>C was associated with EBVs for SCS2 (*P *= 0.038), SCS3 (*P *= 0.045) and over all lactations (*P *= 0.023); the corresponding allele substitution effects for this SNP were -0.103 ± 0.049, -0.112 ± 0.056 and -0.102 ± 0.045, respectively, meaning that allele C reduces SCS over allele A.

**Table 3:** Association of the SNPs in the *SPP1 *gene with EBVs for SCS.

	**Average allelic substitution effect (in EBV units of score)^a^**											
	
	**SCS**			**SCS1**			**SCS2**			**SCS3**		
				
**DNA Polymorphisms**				**Average allele effect ± SE (*P *value)**								
*SPP1c.-1301G>A*	0.047	± 0.030	(0.121)	0.033	± 0.030	(0.280)	0.047	± 0.034	(0.165)	0.035	± 0.038	(0.362)
*SPP1c.-1251C>T*	0.117	± 0.047	(0.014)	0.097	± 0.046	(0.035)	0.118	± 0.052	(0.023)	0.112	± 0.059	(0.056)
*SPP1c.-430G>A*	- 0.117	± 0.047	(0.014)	- 0.097	± 0.046	(0.035)	- 0.118	± 0.052	(0.023)	- 0.112	± 0.059	(0.056)
SPP1c.*40A>C	- 0.102	± 0.045	(0.023)	- 0.068	± 0.044	(0.121)	- 0.103	± 0.049	(0.038)	- 0.112	± 0.056	(0.045)

^a ^EBVs are calculated for the respective lactation: over all (SCS), first (SCS1), second (SCS2), and third (SCS3) lactations

### Effect of *SPP1 *SNPs for production traits

The average substitution effect of the SNP *SPP1c.-1301G>A *was significant for milk yield from the second (*P *= 0.027) and third (*P *= 0.046) lactations. Substitution of allele G over allele A decreased the EBV for milk yield by the equivalent of 178 kg and 165 kg, respectively (Table 4). Although no significant effect was observed for fat or protein yield (kg), the same allele G had a favourable effect on the EBVs for the fat and protein percentages. Protein percentages were associated with the SNP in the second (*P *= 0.030) and third (*P *= 0.036) lactations (0.03% ± 0.01), whereas the effects for fat percentages were observed for all three lactations, as follows: 0.08% ± 0.03 (*P *= 0.004), 0.09% ± 0.03 (*P *= 0.012) and 0.08% ± 0.03 (*P *= 0.009), respectively. None of the three other SNPs was found to have a significant allelic substitution effect when associated with the EBVs for production traits (data not shown) or any significant effect using the regression model (Additional file 1). However, all three SNPs were associated with EBVs for fat yield and fat percentage (*P *< 0.05) (Additional file 1).

**Table 4:** Association of the SNP *SPP1c.-1301G>A *in the *SPP1 *gene with EBVs for production traits.

	**EBV unit**											
	
	**over all lactations**			**1st lactation**			**2 nd lactation**			**3 rd lactation**		
				
**Trait**	**Average allele effect ± SE (p value)**											
Milk yield (Kg)	-116	± 78	(0.142)	-177	± 91	(0.052)	-178	± 80	(0.027)	-164	± 82	(0.046)
Fat yield (Kg)	2	± 3	(0.500)	3	± 3	(0.289)	1	± 3	(0.658)	2	± 3	(0.485)
Protein yield (Kg)	-2	± 2	(0.420)	-3	± 3	(0.175)	-3	± 2	(0.167)	-3	± 2	(0.251)
Fat %	0.05	± 0.03	(0.059)	0.09	± 0.03	(0.004)	0.08	± 0.03	(0.012)	0.08	± 0.03	(0.009)
Protein %	0.02	± 0.01	(0.090)	0.02	± 0.01	(0.060)	0.03	± 0.01	(0.030)	0.03	± 0.01	(0.036)

### Haplotype analysis

Table 5 shows the estimated population haplotype frequencies, comparing two different algorithms provided by the HAPROB and Haploview analysis methods. Both methods reported that four haplotypes were more likely to be present in the population tested. However, HAPROB analysis was more sensitive for detecting low abundant alleles. Block H1 (GCGA) was the most frequent haplotype (59.2%), whereas block H5 was detected in only six offspring among the 578 bulls. Blocks H2, H3 and H4 had frequencies equal to 0.24, 0.10 and 0.05, respectively. The remaining haplotypes, H6 to H13, had frequencies less than 0.2% and were pooled together (into H6) for statistical analysis. Table 6 reports the genotypes of both alleles from all 578 bulls, displayed using the haplotype blocks present in the population (Table 5). The EBVs (least mean squares) for all traits are reported, according to their homozygosis or heterozygosis, sorted by increasing value of the EBV for SCS for the over all lactations. Analysis revealed statistically significant differences between haplotype groups at a comparison-wise level with EBVs for SCS1 (*P *= 0.012), SCS2 (*P *< 0.001), SCS3 (*P *< 0.001) and over all lactations (*P *< 0.001). For production trait, analysis revealed statistically significant differences between haplotype groups at a comparison-wise level with EBVs for fat (*P *= 0.044) and fat percentage (*P *= 0.043) (Table 6), and for protein percentage for the first and second lactations only (data not shown). None of the haplotypes produced a significant allele substitution effect on EBV for SCS or for the production traits (Table 7 or Additional file 1).

**Table 5:** Estimated haplotype block and population frequencies of the *SPP1 *locus.

**Haplotype**	***SPP1c.-1301G>A***	***SPP1c.-1251C>T***	***SPP1c.-430G>A***	SPP1c.*40A>C	**Frequency^a^**	**Frequency^b^**
H1	G	C	G	A	0.59230	0.599
H2	G	T	A	C	0.23976	0.252
H3	A	C	G	A	0.10267	0.105
H4	G	C	G	C	0.05079	0.045
H5	G	T	A	A	0.00704	
H6	A	T	A	C	0.00164	
H7	G	T	G	C	0.00125	
H8	G	C	A	C	0.00123	
H9	A	C	G	C	0.00107	
H10	G	C	A	A	0.00106	
H11	G	T	G	A	0.00104	
H12	A	C	A	C	0.00003	
H13	A	T	G	A	0.00003	
H14	A	T	G	C	0.00003	
H15	A	C	A	A	0.00003	
H16	A	T	A	A	0.00003	

^a^Analysis were performed using HAPROB [72]

^b^Analysis were performed using Haploview [73], version 4

**Table 6:** Estimated haplotype frequencies and effects of haplotypes in the bovine *SPP1 *gene on EBVs for SCS^a ^and for production traits.

**Genotype**			**Least squares mean**								
		
**Allele 1**	**Allele 2**	**Frequency (%)**	**SCS**	**SCS1**	**SCS2**	**SCS3**	**Milk yield (Kg)**	**Fat yield (Kg)**	**Protein yield (Kg)**	**Fat %**	**Protein %**
H4 (GCGC)	H4 (GCGC)	2 (0.4)	2.81	2.9	2.74	2.79	-594	0	-17	0.22	0.02
H1 (GCGA)	H4 (GCGC)	30 (5.2)	2.87	2.90	2.85	2.88	-238	-6	-3	0.03	0.04
H2 (GTAC)	H4 (GCGC)	13 (2.2)	2.88	2.88	2.92	2.88	-333	-8	-8	0.04	0.03
H1 (GCGA)	H2 (GTAC)	170 (29.4)	2.94	2.96	2.92	2.94	-128	1	-0.7	0.07	0.04
H3 (ACGA)	H4 (GCGC)	5 (0.9)	3.01	3.04	3.03	2.96	296	25	8	0.15	-0.01
H1 (GCGA)	H1 (GCGA)	205 (35.6)	3.02	3.00	3.01	3.05	42	-2	1	-0.02	0.001
H2 (GTAC)	H2 (GTAC)	37 (6.4)	3.02	3.02	3.00	3.01	-99	-13	-2	-0.09	0.02
H4 (GCGC)	H5 (GTAA)	6 (1.0)	3.03	3.01	3.03	3.05	-244	4	-8	0.14	0.005
H1 (GCGA)	H3 (ACGA)	76 (13.2)	3.16	3.09	3.13	3.17	-139	2	0.1	0.07	0.05
H2 (GTAC)	H3 (ACGA)	28 (4.8)	3.17	3.15	3.18	3.2	-198	-7	-2	0.01	0.04
H3 (ACGA)	H3 (ACGA)	6 (1.0)	3.38	3.25	3.39	3.50	-517	-11	-10	0.08	0.07
		
		± SEM	± 0.24	± 0.22	± 0.26	± 0.28	± 567	± 19	16	± 0.21	± 0.08
		
		*P *value	<.001	0.012	<.001	<.001	0.236	0.044	0.745	0.043	0.078

^a ^EBVs are calculated for the respective lactation: over all (SCS), first (SCS1), second (SCS2), and third (SCS3) lactations

**Table 7:** Estimated haplotype effects on EBVs for SCS.

	**Average haplotype substitution effect (in EBV units of score)^a^**											
	
**Haplotypes**	**SCS**			**SCS1**			**SCS2**			**SCS3**		
				
**Estimated haplotype effect ± SE (*P *value)**												
H1 (GCGA)	0.118	± 0.268	(0.660)	0.034	± 0.261	(0.896)	0.120	± 0.295	(0.685)	0.173	± 0.333	(0.604)
H2 (GTAC)	0.132	± 0.274	(0.629)	0.061	± 0.266	(0.818)	0.136	± 0.300	(0.650)	0.174	± 0.340	(0.609)
H3 (ACGA)	0.169	± 0.277	(0.541)	0.071	± 0.270	(0.793)	0.177	± 0.305	(0.562)	0.209	± 0.345	(0.545)
H4 (GCGC)	0.023	± 0.276	(0.935)	0.030	± 0.269	(0.913)	0.025	± 0.303	(0.933)	0.071	± 0.343	(0.836)
H5 (GTAA)	0.212	± 0.352	(0.547)	0.090	± 0.339	(0.790)	0.247	± 0.382	(0.518)	0.285	± 0.433	(0.512)

^a ^EBVs are calculated for the respective lactation: over all (SCS), first (SCS1), second (SCS2), and third (SCS3) lactations

## Discussion

The objective of this work was to identify one candidate of the innate immune response, to define the association of the genetic variants with the immune cells in milk, but also to evaluate their impact on other traits related to dairy production. Osteopontin has been described as an early component of the T cell activation mechanism and was also detected in the immune cells in the present study during the first hours of *E. coli *infection. This confirms the results of other studies which show the importance of osteopontin in inflammatory responses. The expression of *SPP1 *is enhanced in T cells during bacterial infection in order to recruit macrophages to the infection site and to improve cell-mediated immunity (Th1) by increasing the secretion of Th1 cytokines [34-36]. Indeed, knockout *SPP1*^-/- ^mice have shown significantly impaired Th1 immunity to viral and bacterial infections with diminished production of interleukin-12 (IL-12) and interferon-gamma (IFN-γ) and elevated production of interleukin-10 (IL-10) [44]. However, no studies have been carried out to correlate osteopontin with mastitis.

Innate immune responses are activated by a cross-species conserved signalling pathway. Study of this activation could lead to the identification of candidates genes for disease resistance. The detection of favourable allelic variants of the innate immune response genes will make it possible to select progeny with a more efficient immune system. Because of the potential immunological role of *SPP1 *in the milk of cows diagnosed with mastitis, the next step was the validation of *SPP1 *genetic variants that would explain the SCS observed in the dairy population. Indeed, although SCS in milk is an indirect measure of cow health status, SCS is considered to be the most widely used biological marker of clinical mastitis in lactating cows [30]. We thus found polymorphisms in the genomic sequence of *SPP1 *that influence the number of somatic cells in milk and, potentially, influence their susceptibility to mastitis, although this latter assumption remains to be validated. Through the use of animals with extreme EBV for SCS, a novel SNP was discovered in the promoter region of *SPP1 *(*SPP1c.-1301G>A*), 50 nucleotides (nt) upstream from the SNP *SPP1c.-1251C>T*.

The 5' UTR SNP *SPP1c.-1251C>T *and *SPP1c.-430G>A*, and the 3' UTR SNP SPP1c.*40A>C were also identified both in the present study and in a previous one [43]. These DNA polymorphisms were not investigated for an association with SCS. However, these authors found one DNA polymorphism (T_9_/T_10 _INDEL) that had an effect on the fat and protein percentage traits, based on the results of 167 bull sires, but was not associated with SCS [43]. This INDEL polymorphism, which corresponds to polyT tract alleles of either nine or 10 thymines (T_9_/T_10 _INDEL), is absent from the 100 bulls sequenced in the present study, and also from the mouse [45], human and swine *SPP1 *promoter (see sequence alignment in Additional file 2A). The bovine *SPP1 *region containing this polymorphism (also -1,301 and -1,251 nt) was aligned with other mammals using the CLUSTALW algorithm (Additional file 2A). This region does not contain any transcription factor (TF) motifs (data not shown). The T_9_/T_10 _INDEL polymorphism may not associate with TF. It is located 1,119 nt upstream from the novel *SPP1c.-1301G>A*, is absent from sequenced individuals selected, and thus may not be a functional SNP for the SCS trait. Therefore, we cannot exclude this T_9_/T_10 _INDEL polymorphism from the Canadian population, since the present experimental design does not address differences among bulls with extreme EBVs based on production traits (e.g. fat).

**Figure 2:**
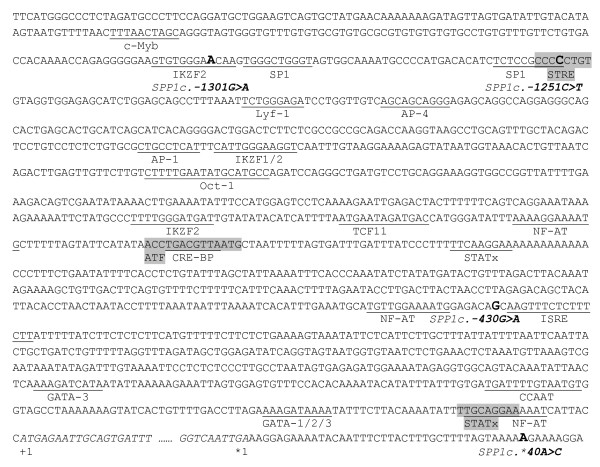
**Nucleotide sequence of the 5' upstream region (GenBank accession No. **AY878328) **of the bovine osteopontin gene *SPP1***. The 5' region is indicated in uppercase letters, and position +1 (translation initiation codon) is indicated in italic uppercase letters until position *1 (translation termination codon). SNPs are in bold. Putative binding sites (found with ) are shown in grey or underlined when co-localized: octamer factor 1 = Oct-1; TCF11/KCR-F1/Nrf1 homodimers = TCF11; LyF-1 = Lyf-1; c-Myb = c-Myb; stimulating protein 1 = SP1; stress-response element = STRE; AP-1 binding site = AP-1; activator protein 4 = AP-4; IKAROS family zinc finger 1 (Ikaros) = IKZF1; IKAROS family zinc finger 2 (Helios) = IKZF2; nuclear factor of activated T-cells = NF-AT; CRE-binding protein 1/c-Jun heterodimer = CRE-BP; signal transducers and activators of transcription = STATx; C/EBPalpha CCAAT/enhancer binding protein alpha = CCAAT; GATA-binding factor 1 = GATA-1; GATA-binding factor 2 = GATA-2; GATA-binding factor 3 = GATA-3; interferon-stimulated response element = ISRE; activating transcription factor = ATF.

We searched for cross-species similarity in regions of the genome containing the SNP detected in the present study. Both of the SNPs in the promoter are very similar to other species (Additional file 2: B and C). Interestingly, these regions included a "GC-rich" area identical to both the mouse and the human regions. Among the potential functional factors that could explain the impact on the EBV for the SCS, IKZF2 (IKAROS family zinc finger 2) and SP1 are two TFs that recognize binding sites at or in the vicinity of the SNP (Figure 2). The TF SP1 binds specifically to GC box DNA within the 5' flanking promoter sequences for promoting eukaryotic transcription [46,47]. Specifically, the TF SP1 binds to the *SPP1 *promoter and is involved in Th1 immune responses [48]. The TF IKZF2 is a regulator of Th2 responses [49]. In that way, allele G of *SPP1c.-1301G>A *abolishes IKZF2 recognition (Figure 2). Thus, impaired IKZF2 binding would have the consequence of promoting a Th1 immune response in the absence of a Th2 polarizing effect on naive immune cells [49]. Impaired IKZF2 binding could also facilitate the access of SP1 (Th1 response) through its binding activity, which is present in the vicinity of *SPP1c.-1301G>A *(Figure 2). This hypothesis is supported by the fact that allele A is associated with an elevated SCS (Table 3), SCS having been associated with increased mastitis incidence [30]. Indeed, an inefficient Th1 response or abrogated innate immunity predisposes cows to environmental mastitis.

An additional SP1 recognition site was found in the location of *SPP1c.-1251C>T*, with allele T abolishing the SP1 GC box recognition (Figure 2). This TF binding site has been found to colocalize with the human "-66T" SNP, which is referred to as the human *SPP1 *transcription starting site [48]. The authors, Hummelshoj et coll., confirmed by electrophoretic mobility shift assay that recognition of the TF SP1 is influenced by the presence of DNA polymorphism. Furthermore, using a luciferase assay reporting the *SPP1 *promoter, they observed increased activity with allele T compared to allele C for the corresponding -66 T/G SNP [48]. Alignment of human and bovine sequences revealed that the -66 T/G SNP is located 7 nt from the bovine SNP *SPP1c.-1251C>T*, both within the TF SP1 binding site. Although it remains to be confirmed, the influence of the SNP *SPP1c.-1251C>T *on the regulation of the bovine promoter is highly plausible. Indeed, the regulation of promoters based around the Sp family binding protein has been observed in a number of housekeeping genes. These factors act as transcriptional activators in mammalian cells [50]. Since SP1 recruits the basal transcription machinery and controls the rate of transcription [51,52], it thus explains the constitutive secretion of osteopontin in several biological fluids (blood, semen, serum, bile) and its distribution in a very broad variety of tissues [53]. Our results suggest that the absence of the SP1 DNA binding site would impair or reduce the transcription activity of the *SPP1 *promoter. Unless validation of the *SPP1 *transcript or osteopontin protein from cows harbouring allele C or T *SPP1c.-1251C>T *is performed (haplotypes H2 and H5 contained an abolished SP1 binding site [Table 6]) the veracity of the tangible impact of *SPP1c.-1251C>T *on bacterial infection remains unknown. Further study by *in vitro *assay using reporter genes and binding shift assays could help elucidate the impact of promoter variants on transcription activity.

Lastly, two other binding sites, the nuclear factor of activated T cells (NF-AT) and the interferon-stimulated response element (ISRE), were positioned close to *SPP1c.-430G>A *(-7 and +2 nt, respectively), located in the first intron. The NF-AT promotes cell proliferation and the expression of inflammatory cytokines, such as *IL-6 *and *SPP1 *[54], whereas ISRE would regulate the expression of *SPP1 *[55]. A polymorphism in this region could potentially affect the binding of these two TFs and influence the rapidity of the response of *SPP1 *to infection. Therefore, functional studies are required to interrogate the significance of this SNP with TF binding and gene expression.

In an association study, neither *SPP1c.-1251C>T *nor *SPP1c.-430G>A *was found to be associated with SCS (Table 2). We found that these two are linked (i.e. C/G or T/A on the same allele; data not shown), which could explain why only heterozygous animals (CT genotype for *SPP1c.-1251C>T *and GA genotype for *SPP1c.-430G>A*), when compared to their respective homozygotes, had an impact on SCS. This was also shown by their opposite allelic substitution effects (Table 3). Therefore, both allele C for *SPP1c.-1251C>T *and allele A for *SPP1c. 430G>A*, which are linked to G and T, respectively, would be required to compensate for the negative effect of the second allele.

As innate immunity is not an adaptive immune response, an association study regarding the genetics should not be modulated as time passes. In other words, immune response should be maintained, irrespective of the number of times the pathogen is encountered. Indeed, this constant trend (for statistically significant values) was observed regarding the allele substitution effect (Table 3), which means that cows harbouring the favourable allele do not benefit (i.e. there is no decrease of the SCS value) in later parities from these *SPP1c.-1251C>T *and *SPP1c.-430G>A *SNPs. The SNP *SPP1c.-1301G>A *was only found to be associated with SCS in the regression model (Table 2). However, it may also have an impact that was not revealed by the allele substitution effect. Because of the discrepancy in the abundance of both alleles (89.5% versus 10.5%; Table 3), the substitution effect may not be statistically significant.

For the fourth SPP1c.*40A>C SNP, it is less clear how the 3' UTR DNA polymorphisms affect *SPP1*, although a more general mechanism such as microRNA would affect transcript stability. We cannot exclude that *SPP1 *would be relatively more stable than other transcripts in milk cells. Although amplitude of the *SPP1 *induction was smaller than *TNF *transcript, both were correlated as the bacterial challenge of the PBMC progressed (data not shown). The TNF-α is an important inflammatory mediator involved in neutrophil recruitment [56,57] and *SPP1 *might activate or maintained the Th1-Th2 balance. Thus *SPP1 *could be either an upregulated gene or stabilized transcript. MicroRNA are an important class of regulatory RNA that repress animal genes by preferentially interacting with complementary sequence motifs in the 3' UTR of target mRNA [58]. The importance of the region is further highlighted by the presence of the SPP1c.*40A>C SNP within two other bovine species. The presence of this DNA polymorphisms was detected within the Guernsey (n = 42) and Jerseys (n = 81) cows (data not shown). Whereas both human and mouse 3' UTR of *SPP1 *are the target of several microRNA, retrieved by an *in silico *search (; data not shown), this hypothesis remains to be verified for the bovine *SPP1 *3' UTR sequences.

In previous studies, nine DNA polymorphisms were found within eight sires sequenced for the *SPP1 *gene. Whereas one SNP was associated with production traits (fat and protein yield and percentage, milk yield), this "*OPN3909*" was not found associated with SCS [43]. The main reason may be inherent to the selection of the Holstein dairy cattle population, which in our study was based on extreme EBV for SCS from a directory of 6,453 bulls (Canadian Dairy Network database). How *SPP1 *may affect fat yield and fat percentage in bovine milk remains an open question. In humans, a significant positive correlation was found between osteopontin levels and body fat as well as circulating concentrations of total cholesterol [59]. Osteopontin is also associated with cholesterol gallstone formation in human and mouse [60]. The specific role of osteopontin beyond its immune role in milk warrants further investigation. From a genetic perspective, it is well known that health and disease may affect other performance traits such as longevity and fertility among others. Therefore, genetic factors affecting health trait may impact other traits. For bull fertility, the EBV for the male fertility index is reported as the non-return rate trait (Canadian Dairy Network database). Among the genotyped bulls, we did not find significant statistical association of the four *SPP1 *SNP with the EBV of the non-return rate (data not shown). Even though osteopontin was reported to influence bull fertility [61,62], these *SPP1 *variants were not found associated with male fertility (data not shown). We cannot exclude that a genetic association could be observed using bulls with extreme EBV for the male fertility index. Therefore, further investigations are still required before suggesting favourable allele for the innate immune system, such as how the genetic *SPP1 *variants may influence male fertility or milk composition (e.g. concentration of cholesterol). In fact, milk is used for human consumption and an increase of the concentration of cholesterol may not be valuable.

In the present study, we found a strong correlation between *SPP1 *and SCS, and we identified potential functional sites in the promoter of the gene. The different promoter haplotypes have been cloned, and the relevance of the functional sites will be studied in order to elucidate the molecular mechanisms responsible for the abundance of milk somatic cells in the Holstein dairy population as well as to depict the immune response to environmental stimuli.

## Conclusion

This study reports the link between DNA polymorphisms within the innate immune *SPP1 *gene, the number of milk immune cells and, potentially, susceptibility to mastitis. Indeed, one SNP abolished the SP1 recognition site, whereas another SNP affected the transcription binding factor IKAROS. These SNPs, located in the promoter, are potentially involved in the Th1 immune response and in the Th2 regulation pathway since they were identified by in silico search to abolish these transcription factor recognition sites. Because these SNPs are associated with the level of immune (somatic) cells measured in milk which is associated with clinical mastitis and because of their potential implication in the Th1- Th2, all together, these findings suggest the genetic potential of these variants in terms of selection for improving mastitis resistance in dairy cows. But osteopontin is not only involved in regulatory mechanisms of the immune response but also in mammary gland development and milk secretion. Thus, before recommending any *SPP1 *haplotype for genetic selection, the beneficial role of these genetic forms would require further investigation.

## Methods

### Animals

Twenty multiparous Holstein cows were selected from surrounding dairy farms based on several criteria. All cows were from different dam and sire families, and had low SCC ranging from 5 × 10^4 ^to 1 × 10^5 ^cells/mL, no past history of mastitis, tested negative for bovine virus diarrhoea, leucosis, neospora and mycoplasmosis, and were found to be free from bacteria in aseptically collected milk samples. Milk was aseptically sampled from each quarter every week starting at calving until the end of the trial period (110 to 170 days of lactation). Four cows were kept for the study based on these criteria: no calving associated problem and bacteriologically negative milk samples with SCC values below 1 × 10^5 ^cells/mL across lactation. The cows were transferred to the level 2 biosecurity facility one week before the challenge. They were handled according to the Guide for the Care and Use of Agricultural Animals by the Institutional Animal Care and Use Committee at Agriculture and Agri-Food Canada's Dairy and Swine Research and Development Centre (Document 188).

### Bacteria and intramammary challenge

*Escherichia coli *strain SHY97-3923-2, previously isolated from a clinical case of bovine mastitis and kindly provided by the Laboratoire provincial de pathologie animale (St-Hyacinthe, Quebec, Canada), was used for the intramammary challenge. An antibiotic susceptibility test was validated on the strain (Biovet, St-Hyacinthe, Quebec, Canada). The bacteria were cultivated in tryptic soy broth (TSB) and the fresh starter was incubated for approximately 3 h to bring it to an exponential growth phase (0.6 to 0.7 OD). The culture was then centrifuged, washed once in pyrogen-free saline (PFS) and re-suspended following an established serial dilution protocol to get a final concentration of 1,000 CFU/mL. The concentration was confirmed by plating 200 μL of three independent dilutions on TSA. The 3-mL volume was injected in the challenged glands via the teat canal immediately after the morning milking in the left and right rear quarters of the mammary gland (n = 4 cows). As controls, the right and left front quarters of each cow were infused with 3 mL of sterile saline solution. All the quarters were then massaged to ensure dispersal of the inoculum. Bacteriological analysis was conducted by plating 200 μL of each milk sample from each quarter onto TSA overnight at 37°C to determine the number of colony-forming units over the challenge.

### Clinical signs, collected samples and assays

Antibiotic treatment (Borgal; Hoechst, Montreal, Canada) and pain relief medicine (Anafen; Merial, Baie d'Urfé, Canada) were administered following 6 h (n = 2 cows) and 12 h (n = 2) according to the Guide for the Care and Use of Agricultural Animals. Rectal temperature and general state were monitored for each cow every 3 h. The SCC in milk samples were analyzed by the Dairy Production Centre of Expertise (Valacta, Montreal, Quebec, Canada) and provided values on crude milk. All contaminated samples were processed in the level 2 biosecurity microbiology laboratory. The tumour necrosis factor-alpha (TNF-α) concentration in the milk samples was determined using an enzyme-linked immunosorbent assay (ELISA) kit provided by the Vaccine and Infectious Disease Organization (Saskatoon, Saskatchewan, Canada). The assay was performed according to the protocol developed at the Vaccine and Infectious Disease Organization and as described previously by other authors [63].

### Fluorescence-activated cell sorting

Measurement of differential leukocyte populations in milk was performed by flow cytometry using the SYTO 13 labelling method, as described previously by other authors [64] but with some modifications. Briefly, 15 mL of collected milk was added to 35 mL of 1× Hank's balanced salt solution without Ca^2+ ^or Mg^2+ ^(HBSS 10×; Invitrogen, Toronto, Ontario, Canada) and centrifuged. The pellet was re-suspended in RPMI-1640 (Invitrogen) supplemented with 5% fetal bovine serum (FBS; Invitrogen) at 1 × 10^5 ^to 1 × 10^6 ^cells/mL. Then, 490 μL was transferred to a new tube, and 10 μL of diluted SYTO 13 green fluorescent nucleic acid stain solution (1:400 in RPMI-1640; 5 mM, Invitrogen) was added. After 10 min in the dark, the staining was stopped with 4 mL of HBSS 1×, and the cells were recolted. The pellet was re-suspended in 400 μL of cold RPMI-1640 supplemented with 5% FBS, and was analyzed with a Coulter Epics XL-MCL flow cytometer using Expo 32 software (Beckman Coulter, Mississauga, Ontario, Canada). The forward scatter and side scatter were measured on a linear scale, whereas green fluorescence was registered on a log scale. The differential leukocyte count in milk after SYTO 13 staining was quantified using the side scatter/green fluorescence dot plot. The percentage of different leukocyte populations in milk samples was established, after counting 10,000 events, as follows: percentage of the number of cells in the gated leukocyte population out of the total number of gated lymphocytes, macrophages and granulocytes.

### RNA extraction and analysis

Mammary quarters were sampled aseptically. Twenty mL was diluted in an equal volume of PBS and centrifuged at 180 × g for 10 min at 4°C. The cell pellet was washed with cold PBS and 1 mL of TRIzol (Invitrogen, Carlsbad, California, USA) was added. The RNA extraction was performed as recommended by the manufacturer but with minor modifications. The RNA samples were resuspended in water with SUPERase. In (1 U/μL; Ambion) and treated with Recombinant DNase I (Ambion). The final concentration was determined using a NanoDrop ND-1000 spectrophotometer. For each cow, equal amounts of RNA from the infected rear quarters and the non-infected forequarters were pooled, with one pool for the infected quarters and one for the non-infected quarters. RNA was amplified using the SMART mRNA Amplification Kit (Clontech, Mountain View, California, USA) according to the manufacturer's protocol. This kit uses the template-switch mechanism to generate the double-stranded cDNA necessary for *in vitro *transcription without the polymerase chain reaction (PCR) step that is generally associated with a conventional SMART technique. The cDNA library was performed according to the recommended protocol for the PCR-Select cDNA Subtraction Kit (Clontech), which is a suppressive subtractive hybridization (SSH) technique. Briefly, equal amounts of amplified RNA from both infected quarters of cows were pooled prior to the hybridizations. The same pooling step was performed for the non-infected quarters. The pool of infected samples was used as the "tester" in accordance with the SSH procedures and was subtracted with an excess of the "driver," the "driver" being the pool prepared from transcripts derived from the non-infected quarters. Consequently, this scheme allows the generation of a differential representation of infected transcripts normalized with non-infected transcripts. Supplementary PCR amplifications of the hybridization product were then cloned into a TA vector (pCRII, Invitrogen) and transformed into MAX Efficiency DH5α-competent cells (Invitrogen) to make up a cDNA library. To identify clones containing a single insert, each clone was submitted to PCR using NP1 and NP2R primers, and the products were electrophoresed on a 2% agarose gel. Clones were sequenced according to the BigDye Terminator v3.1 Cycle Sequencing Kit protocol with a 3100-Avant genetic analyzer (Applied Biosystems, Foster City, California, USA). Lastly, gene similarity was searched among our sequences using the Basic Local Alignment Search Tool (BLAST) algorithm of the National Center for Biotechnology Information (NCBI) public database.

### Isolation of bovine PBMC and induction

Peripheral blood mononuclear cells (PBMC) were isolated from the buffy coat fractions of peripheral blood and further purified by Ficoll and sucrose gradients. Blood was collected from the jugular or caudal vein in 10 × heparin (1:10) tubes (n = 3 cows). Centrifugation was performed at 572 × g for 15 min at 18°C. For the respective cow, the fractions were pooled together, completed to 40 mL with 1× HBSS (Hanks' Balanced Salt Solution without Ca^2+ ^and Mg^2+^; Wisent, St-Bruno, Canada), poured very slowly onto 2 × Ficoll gradients (Ficoll Paque Plus; Amersham, Baie d'Urfe, Canada), and centrifuged at 572 × g for 40 min at 18°C. The PBMC were rinsed with 1× HBSS, re-suspended in 5 mL of 1× HBSS and poured onto a 20% sucrose (Sigma, Oakville, Ontario) discontinous gradient prior to centrifugation at the same parameters described above. The PBMC were rinsed with 1× HBSS and the cell pellets were treated with Red Cell Lysis buffer (Sigma) to eliminate residual erythrocytes. Finally, PBMC were re-suspended in 2 mL RPMI-1640 (Wisent) with 5% foetal calf serum (Wisent). Cells were cultured at 5 x 10^6 ^in 12 wells flat bottom plate at 39°C in 5% CO_2 _in a humidified atmosphere. Cells were incubated for 45 min to allow monocytes to attach before bacterial induction and incubated with either medium alone (nonstimulated) or with heat-inactivated bacteria (63°C for 30 min, as described before [65]) at a concentration of 30 CFU/PBMC. The samples were harvested immediately after the attachment period (time zero) or after 0.5, 1, 3 or 5 h post-infection. Cells in suspension were harvested, 1 mL of TRIzol was added to each well and transferred to the respective pellet cells in order to recover both attached and cells in suspension. The RNA extraction was performed as described above.

### RT-PCR and quantitative RT-PCR

The reverse transcription (RT) was performed with 1 μg of RNA according to the SuperScript II RT procedure (Invitrogen). Each RT assay was made in a 20-μl reaction using Oligo(dT)_12-18 _(Invitrogen) as a primer and according to the supplier's recommendations. Quantitative RT-PCR (qRT-PCR) was performed in a 10-μL final volume using 5 μL of Fast SYBR Green Master Mix 2× (Applied Biosystems), 300 nM of both forward and reverse primers (Additional file 3), and 2 μL of diluted template. All RT reactions were performed in triplicate. The amplification was carried in a 7500 Fast Real-Time PCR System (Applied Biosystems) following denaturation of 20 s at 95°C and amplification during 40 cycles of denaturation at 95°C for 3 s followed by an annealing/elongation period of 20 s at 60°C. Three reference genes were also measured, namely actin beta (*ACTB*), glyceraldehyde-3-phosphate dehydrogenase (*GAPDH*) and peptidylprolyl isomerase A (*PPIA*). The qRT-PCR results were analyzed according to the relative quantification method given by the arithmetic formula 2^-ΔΔCt ^[66].

### Genetic DNA material and EBVs for the SCS and milk production traits

Selection of Holstein genetics was performed using information downloaded from the Canadian Dairy Network database (Guelph, Ontario, Canada), which can also be accessed using the web interface . The EBV data for the August 2008 genetic evaluation were downloaded onto a local server. These EBVs comprised the SCS, the non-return rate, and production (protein and fat yields, protein and fat percentages, and milk yield) over lactations. The semen of 578 bulls presenting a mean EBV of 3.01 (Table 8) for the SCS, which is very close to the breed average (3.00; Canadian Dairy Network database), was available for the study. Table 8 describes the EBVs (SCS) for the different lactations. Among the 6,453 recorded bulls, two selections were made using the 150 lowest-ranked and 150 highest-ranked bulls for the EBV for SCS. Among these two groups, semen was available for 51 and 50 bulls, corresponding respectively to the low and high cohorts for the EBV (SCS), and thus defined as the low SCS (2.48 ± 0.07) and the high SCS (3.72 ± 0.17) pools. Genomic DNA (gDNA) was extracted from semen samples, as described previously by other authors [67] and concentration was measured using the NanoDrop ND-1000 spectrophotometer.

**Table 8:** Descriptive statistics of the EBVs for the SCS trait for the Holstein bulls^a^.

**EBV^b ^units**					**Bulls selected for SNP mining**							
					
	**Bulls' cohort**				**Low EBV for SCS**				**High EBV for SCS**			
			
	**Nb**	**Mean**	**± SD**	**[min-max]**	**Nb**	**Mean**	**± SD**	**[min-max]**	**Nb**	**mean**	**± SD**	**[min-max]**
SCS	578	3.01	± 0.35	[2.34-4.16]	51	2.48	± 0.07	[2.34-2.72]	50	3.72	± 0.17	[2.99-4.16]
SCS1	521	3.00	± 0.31	[2.36-4.36]	49	2.53	± 0.08	[2.36-2.65]	45	3.63	± 0.18	[3.37-4.36]
SCS2	521	3.00	± 0.37	[2.28-4.12]	49	2.47	± 0.09	[2.29-2.62]	45	3.76	± 0.15	[3.40-4.12]
SCS3	521	3.03	± 0.41	[2.08-4.15]	49	2.42	± 0.13	[2.13-2.70]	45	3.82	± 0.19	[3.42-4.26]
	
	Breed average: 3.00				Selected sub-group average: 3.09							

^a ^EBV values from the Canadian Dairy Network database, evaluation published on August 2008

^b ^EBVs are calculated for the respective lactation: over all (SCS), first (SCS1), second (SCS2), and third (SCS3) lactations.

### SNP mining and sequencing

A sequence of 12,300 bp (GenBank accession No. AY878328) was retrieved by a BLAST search for similarity to a consensus sequence assembled from the *SPP1 *transcript sequences from our cDNA library. We used the multiple sequence alignment methods (CLUSTALW) of the DNASTAR suite program MegAlign (Lasergene, v7.2; DNASTAR, Madison, Wisconsin, USA) (data not shown). The sequence of the 5' region of *SPP1 *was analyzed *in silico *for identification of transcription factors' recognized DNA binding motifs (Figure 2) using the software MOTIF Search  with a cut off score of 85. To construct both pools (high and low EBV for SC) for SNP detection, equal amount of each bulls was PCR-amplified for the respective delimited genomic sequence of *SPP1 *(promoter or respective exons) using the appropriate primers designed from the AY878328 sequence (Additional file 4). PCR reactions were carried out in a final volume of 50 μL containing 2 ng of template, 200 nM of each primer, 200 μM of each dNTP, 1.5 mM of MgCl_2_, 1× PCR buffer (20 mM of Tris-HCl pH 8.4, 20 mM of KCl) and 1 U of Taq DNA polymerase (BioShop Canada, Burlington, Ontario, Canada). The conditions were 94°C for 3 min, followed by 35 cycles of 30 s of denaturation (94°C), 30 s of annealing (59°C) and 1 min of elongation (72°C), followed by 5 min of final elongation (72°C). The amplicon size was confirmed by 1.2% agarose gel electrophoresis. The amplified fragments were purified using NucleoFast PCR plates (Macherey-Nagel distributed by MJS BioLynx, Brockville, Ontario, Canada), quantified with the NanoDrop and diluted to a final concentration of 10 ng/μL. To construct the selective DNA pools, equal amounts of the amplified genomic fragments from each bull were assembled into the respective high and low EBV (SCS) pools. Two pools comprising different individuals were assembled, one each for the high and low EBV (SCS) DNA. These pools were used as templates for sequencing with the BigDye Terminator v3.1 Cycle Sequencing Kit (Applied Biosystems) according to the company's recommendations, using the forward or reverse PCR primer (Additional file 4) to sequence both strands. When a SNP was detected (overlap of nucleotide bases), all bulls from the pool were individually sequenced to confirm the DNA polymorphisms.

### SNP genotyping

Five hundred seventy-eight bulls, from 26 different sires, were genotyped using tetra-primer amplification refractory mutation system PCR (ARMS-PCR), as described previously by other authors [68-70]. Primers designed using the open source Cedar Genetics Software  are listed in Additional file 5. Reactions were carried out in a 25-μL final volume containing 50 ng of template in the presence of the appropriate concentration of primers (Additional file 5), 200 μM of each dNTP, 1.5 mM of MgCl_2_, 1× PCR buffer (20 mM of Tris-HCl pH8.4 and 20 mM of KCl), and 1 U of Taq DNA polymerase (BioShop Canada). The PCR reactions were performed at 94°C for 3 min, followed by 35 cycles of 30 s of denaturation (94°C), 30 s of annealing at the appropriate temperature (Additional file 5) and 1 min of elongation (72°C), followed by 5 min of final elongation (72°C). The genotypes were determined according to the fragment length analyzed following 2% agarose gel electrophoresis, as described previously by other authors [68].

### Statistical analysis

Descriptive statistics for the selective pools (high and low EBV pools for SCS) and the genotyped cohort of 578 bulls are presented in Table 2. Allele and genotype association analyses were done through comparison of the frequencies of the different genotypes and alleles with EBVs for SCS for the respective lactation using logistic regression. Conformance of the allele frequencies with the Hardy-Weinberg equilibrium for all the SNPs was tested using the Chi-square test. All analyses were performed using Statistical Analysis System (SAS) software (release 9.1; SAS Institute, Cary, North Carolina, USA).

The average allele substitution effects of the SNPs were calculated using the model described above [68], as follows:



where Y_jk _is the trait EBV of the j^th ^animal in the k^th ^sire, μ is the overall mean, β_i _is the fixed linear regression coefficient for the i^th ^SNP, G_i _is the genotype of the i^th ^SNP recoded [71] (namely -1 [homozygous dominant/abundant genotype], 0 [heterozygous genotype] or 1 [homozygous recessive genotype]), S_j _is the random polygenic effect of the j^th ^(1 to 26) sire, and e_jk _is the random error. The bull's EBVs for SCS in the first (SCS1), second (SCS2), third (SCS3) and global (SCS) lactations were used.

### Haplotype

The four SNPs detected in the osteopontin gene were used for the construction of haplotype blocks. A total of 578 bulls distributed into 26 Holstein families were selected, thus eliminating families with fewer than 10 offspring. The haplotype reconstruction was performed using the HAPROB algorithm [72], and the results were compared using Haploview (v4.1; [73]). Each family comprised a minimum of 10 half-sib sons, which is considered a minimum family size for reaching greater than 80% accuracy, as reported previously by other authors [72]. From the 578 bulls genotyped, 16 haplotypes were reconstructed, but 11 of those had very low probabilities and were then pooled together to represent a single haplotype for the study on haplotype effect. The linear effects of the six haplotypes were estimated, restricting block H6 to an estimated effect equal to 0 to account for a linear dependency among haplotype effects, as described elsewhere [68]. Haplotype effects were estimated with PROC MIXED of SAS (SAS Institute), using the same model as described above [68], as follows:



where Y_jk _is the trait EBV of the j^th ^animal in the k^th ^sire, μ is the overall mean, β_i _is the fixed linear regression coefficient for the i^th ^haplotype, Hap_ik _is the probability of the i^th ^haplotype for the k^th ^bull, S_j _is the random polygenic effect of the j^th ^(1 to 26) sire, and e_ijk _is the random error. Results were considered significant if *P *values were less than 0.05.

## Authors' contributions

CT and JS participated in the design and carried out the experimentation on the cows (bacterial challenge and sample collection). JS performed the bacterial count, KA and CT performed the molecular experiments. ML's team performed the fluorescence-activated cell sorting assays. NAK's team provided 500 bull DNA samples. NB conceived the study. KA and NB interpreted the results and wrote the manuscript. NAK, ML and CT revised the manuscript. All authors read and approved the final manuscript.

## Supplementary Material

Additional file 1 **Effects of polymorphisms in the bovine *SPP1 *gene on EBVs for production traits**. The data provided represent the statistical analysis of the least mean squares of the estimated breeding values of different traits for the respective DNA polymorphisms *SPP1c.-1301G>A*, *SPP1c.-1251C>T*, *SPP1c.-430G>A*, and SPP1c.*40A>C. Click here for file

Additional file 2 **Sequence alignment of the different regions of *SPP1 *surrounding (A) -	2,419 nucleotides (nt) (T_9_/T_10 _INDEL), (B) -1,301 nt (*SPP1c.-1301G>A*), and (C) -1,251 nt (*SPP1c.-1251C>T*)**. The figure presents the sequence alignment of the respective regions surrounding the three SNPs of the bovine *SPP1*, compared with other mammals using the CLUSTALW algorithm. Click here for file

Additional file 3 **Oligonucleotide primer sequences for quantitative real-time PCR**. Sequence of the primers used in quantitative real-time PCR assays for the detection of *SPP1*, *TNF*, *ACTB*, *GAPDH*, and *PPIA *genes are listed. Click here for file

Additional file 4 **Sequencing primers for *SPP1***. The sequence of the primers designed from the AY878328 sequence and used to amplify the respective delimited genomic sequence of *SPP1 *(promoter or respective exons) are listed. Click here for file

Additional file 5 **Primers and conditions for SNP genotyping using tetra-primer ARMS-PCR in the bovine *SPP1 *gene**. Sequence of the primers and the conditions of the PCR reactions used to genotype *SPP1 *using tetra-primer amplification refractory mutation system PCR (ARMS-PCR) are listed. Click here for file
