# Supplementary material for: Osteopontin: an early innate immune marker of Escherichia coli mastitis harbors genetic polymorphisms with possible links with resistance to mastitis
Source: BMC Genomics. 2009 Sep 18;10:444. doi: 10.1186/1471-2164-10-444 (PMC2761946; doi:10.1186/1471-2164-10-444)
Supplement: Additional file 1 — Effects of polymorphisms in the bovine SPP1 gene on EBVs for production traits. The data provided represent the statistical analysis of the least mean squares of the estimated breeding values of different traits for the respective DNA polymorphisms SPP1c.-1301G>A, SPP1c.-1251C>T, SPP1c.-430G>A, and SPP1c.*40A>C. [file 1471-2164-10-444-S1.DOC]

**Additional file 1.**

| DNA polymorphism | Genotype | Milk yield (Kg) | Fat yield (Kg) | Protein yield (Kg) | Fat % | Protein % |
| --- | --- | --- | --- | --- | --- | --- |
|  |  | Least mean squares | | | | |
| *SPP1c.-1301G>A* | GG | -67 | -2 | -2 | 0.01 | 0.02 |
|  | GA | -134 | 1 | < -1 | 0.06 | 0.04 |
|  | AA | -517 | -11 | -10 | 0.01 | 0.07 |
|  | ± SEM | ± 328 | ± 11 | ± 9 | ± 0.12 | ± 0.05 |
|  | *P* value | 0.304 | 0.410 | 0.602 | 0.288 | 0.074 |
|  |  |  |  |  |  |  |
| *SPP1c.-1251C>T* | CC | -36 | -1^a^ | < 1 | 0.01^a,b^ | 0.02 |
|  | CT | -152 | < -1^a^ | -2 | 0.06^a^ | 0.04 |
|  | TT | -99 | -13^b^ | -2 | -0.09^b^ | 0.02 |
|  | ± SEM | ± 132 | ± 4 | ± 4 | ± 0.05 | ± 0.02 |
|  | *P* value | 0.257 | 0.026 | 0.630 | 0.012 | 0.169 |
|  |  |  |  |  |  |  |
| *SPP1c.-430G>A* | GG | -36 | -1^a^ | < 1 | 0.01^a,b^ | 0.02 |
|  | GA | -152 | < -1^a^ | -2 | 0.06^a^ | 0.04 |
|  | AA | -99 | -13^b^ | -2 | -0.09^b^ | 0.02 |
|  | ± SEM | ± 132 | ± 4 | ± 4 | ± 0.05 | ± 0.02 |
|  | *P* value | 0.257 | 0.026 | 0.630 | 0.012 | 0.169 |
|  |  |  |  |  |  |  |
| *SPP1c.*40A>C* | AA | -17 | -1^a^ | 1 | < .01 | 0.02 |
|  | AC | -144 | < -1^a^ | -1 | 0.06 | 0.04 |
|  | CC | -176 | -11^b^ | -4 | -0.04 | 0.02 |
|  | ± SEM | ± 111 | ± 4 | ± 3 | ± 0.04 | ± 0.02 |
|  | *P* value | 0.135 | 0.022 | 0.352 | 0.031 | 0.110 |

^a,b,c^ Means (within a line) without a common superscript letter differ from each other at the 5% level of significance.
