# Supplementary material for: Osteopontin: an early innate immune marker of Escherichia coli mastitis harbors genetic polymorphisms with possible links with resistance to mastitis
Source: BMC Genomics. 2009 Sep 18;10:444. doi: 10.1186/1471-2164-10-444 (PMC2761946; doi:10.1186/1471-2164-10-444)
Supplement: Additional file 2 — Sequence alignment of the different regions of SPP1 surrounding (A) - 2,419 nucleotides (nt) (T9/T10 INDEL), (B) -1,301 nt (SPP1c.-1301G>A), and (C) -1,251 nt (SPP1c.-1251C>T). The figure presents the sequence alignment of the respective regions surrounding the three SNPs of the bovine SPP1, compared with other mammals using the CLUSTALW algorithm. [file 1471-2164-10-444-S2.DOC]

**Additional file 2**


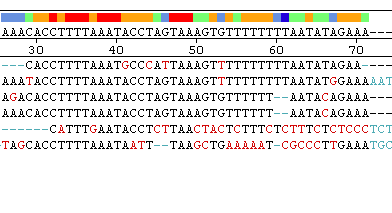


BOVINE ^a1^

CANINE ^b1^

HUMAN ^c1^

CHIMP ^d1^

MOUSE ^e1^

PORCINE ^f1^

**A**


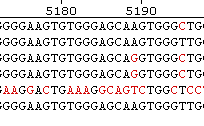


**B**


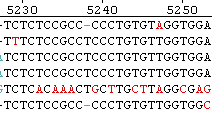


**C**

BOVINE ^a2^

CANINE ^b2^

HUMAN ^c2^

CHIMP ^d2^

MOUSE ^e2^

PORCINE ^f2^

BOVINE ^a3^

CANINE ^b3^

HUMAN ^c3^

CHIMP ^d3^

MOUSE ^e3^

PORCINE ^f3^

^a^Complement of nt 3,878–3,916^1^, nt 5,015–5,035^2^ and nt 5,065–5,085^3^ of GenBank accession No. [AY878328](http://www.ncbi.nlm.nih.gov/nuccore/62084752).

^b^Complement of nt 14,313,006–14,313,044^1^, nt 14,314,352–14,314,372^2^ and nt 14,314,403–14,314,424^3^ of GenBank accession No. [NC_006614](http://www.ncbi.nlm.nih.gov/nuccore/74035448).

^c^Complement of nt 1,130–1,166^1^, nt 2,148–2,168^2^ and nt 2,199–2,220^3^ of GenBank accession No. [D14813](http://www.ncbi.nlm.nih.gov/nuccore/506341).

^d^Complement of nt 90,886,781–90,886,817^1^, nt 90,887,798–90,887,818^2^ and nt 90,887,849–90,887,870^3^ of GenBank accession No. [NC_006471](http://www.ncbi.nlm.nih.gov/nuccore/114796133).

^e^Complement of nt 1,660–1,698^1^, nt 3,039–2,059^2^ and nt 2,092–2,113^3^ of GenBank accession No. [D14816](http://www.ncbi.nlm.nih.gov/nuccore/506348).

^f^Complement of nt 3,339–3,374^1^, nt 2,495–2,515^2^ and nt 2,544–2,564^3^ of GenBank accession No. [M84121](http://www.ncbi.nlm.nih.gov/nuccore/164599).
