# Supplementary material for: Osteopontin: an early innate immune marker of Escherichia coli mastitis harbors genetic polymorphisms with possible links with resistance to mastitis
Source: BMC Genomics. 2009 Sep 18;10:444. doi: 10.1186/1471-2164-10-444 (PMC2761946; doi:10.1186/1471-2164-10-444)
Supplement: Additional file 3 — Oligonucleotide primer sequences for quantitative real-time PCR. Sequence of the primers used in quantitative real-time PCR assays for the detection of SPP1, TNF, ACTB, GAPDH, and PPIA genes are listed. [file 1471-2164-10-444-S3.DOC]

**Additional file 3**. Oligonucleotide primer sequences for quantitative real-time PCR.

| Gene name |  | Primer sequences (5’-3’) | Size bp |
| --- | --- | --- | --- |
| *SPP1* ^a^ | Forward | GCTTCATGGTAACACCCTTCTAAACT | 137 |
|  | Reverse | TGTTTGCTTCTACATGAATTTCTATTCC |  |
| *TNF* ^b^ | Forward | GACGGGCTTTACCTCATCTACTCA | 100 |
|  | Reverse | AGACTGCAATGCGGCTGAT |  |
| *ACTB* ^c^ | Forward | TGGCACCCAGCACAATGA | 123 |
|  | Reverse | CCTGCTTGCTGATCCACATCT |  |
| *GAPDH* ^e^ | Forward | CAGGGTGGTGGACCTCATG | 138 |
|  | Reverse | GGAGATTCTCAGTGTGGCGG |  |
| *PPIA* ^f^ | Forward | ATGCTGGCCCCAACACAA | 101 |
|  | Reverse | CCCTCTTTCACCTTGCCAAA |  |

^a^ Complement of nt 1064 to 1200 of GenBank accession no. [NM_174187](http://www.ncbi.nlm.nih.gov/entrez/viewer.fcgi?db=nuccore&val=31342632)

^b^ Complement of nt 582 to 681 of GenBank accession no.  [NM_173966](http://www.ncbi.nlm.nih.gov/entrez/viewer.fcgi?db=nuccore&val=145279648)

^c^ Complement of nt 1051 to 1173 of GenBank accession no. [NM_173979](http://www.ncbi.nlm.nih.gov/entrez/viewer.fcgi?db=nuccore&val=75832053)

^e^ Complement of nt 1032 to 1169 of GenBank accession no. [NM_001034034](http://www.ncbi.nlm.nih.gov/entrez/viewer.fcgi?db=nuccore&val=77404272)

^f^ Complement of nt 317 to 417 of GenBank accession no. [NM_178320](http://www.ncbi.nlm.nih.gov/entrez/viewer.fcgi?db=nuccore&val=31342068)
