# Supplementary material for: Osteopontin: an early innate immune marker of Escherichia coli mastitis harbors genetic polymorphisms with possible links with resistance to mastitis
Source: BMC Genomics. 2009 Sep 18;10:444. doi: 10.1186/1471-2164-10-444 (PMC2761946; doi:10.1186/1471-2164-10-444)
Supplement: Additional file 4 — Sequencing primers for SPP1. The sequence of the primers designed from the AY878328 sequence and used to amplify the respective delimited genomic sequence of SPP1 (promoter or respective exons) are listed. [file 1471-2164-10-444-S4.DOC]

**Additional file 4**. Sequencing primers for *SPP1*.

| Genomic regions |  | Primer sequences (5’-3’)  For Amplification and Sequencing | Annealing °C | Size bp |
| --- | --- | --- | --- | --- |
| Promoter^a^ | Forward | TGGTGCCAAAGAAAAGTT | 59 | 1033 |
|  | Reverse | GTGCAGCAGCTTGTCATGTAGAC |  |  |
|  | Seq.^h^ | TCTGTCAAATGCAGAGTTCGTGGG |  |  |
|  |  |  |  |  |
| Promoter + Exon 1^b^ | Forward | AGTTATCAGATCCATCAGCTCCAAA | 59 | 850 |
|  | Reverse | CTTACAAATTGACCTTCCCAATGA |  |  |
|  |  |  |  |  |
| Intron 1^c^ | Forward | ATTCTGGGAGATCCTGGTTGTCAG | 59 | 1408 |
|  | Reverse | GCCAGAACTGGTCGGTTTAACCTTTG |  |  |
|  | Seq.^h^ | CCTTGACTTACTAACCTTAGAGACAGC |  |  |
|  | Seq.^h^ | ACTATTAGGCAAGGGAGAGAGG |  |  |
|  |  |  |  |  |
| Exons 2-3 (+intron2)^d^ | Forward | GAGATGGAAAATAGAGGTGGCAGTA | 59 | 545 |
|  | Reverse | AAGCATAGCAGGCACACAATAAAT |  |  |
|  |  |  |  |  |
| Exons 4-5 (+intron4)^e^ | Forward | TTGTCAAGATTGGAGAAGAACAGTTG | 59 | 809 |
|  | Reverse | CGGAATTTGAACCTGGGCAATGTC |  |  |
|  |  |  |  |  |
| Exon 6^f^ | Forward | TGCACTGTAAAGCCTAAGGGACAG | 59 | 436 |
|  | Reverse | GCAGACTCTAGTTTCCTAGAATTGGGC |  |  |
|  |  |  |  |  |
| Exon 7^g^ | Forward | TTACAGTGCTTCCCTTCCTAGCTG | 59 | 974 |
|  | Reverse | ACGGGAACTTTAGCTCAGTCAGAGTC |  |  |

^a^ Complement of nt 3561 to 4593 of GenBank accession no. [AY878328](http://www.ncbi.nlm.nih.gov/entrez/viewer.fcgi?db=nuccore&id=62084752).

^b^ Complement of nt 4448 to 5297 of GenBank accession no. [AY878328](http://www.ncbi.nlm.nih.gov/entrez/viewer.fcgi?db=nuccore&id=62084752).

^c^ Complement of nt 5111 to 6518 of GenBank accession no. [AY878328](http://www.ncbi.nlm.nih.gov/entrez/viewer.fcgi?db=nuccore&id=62084752).

^d^ Complement of nt 6123 to 6667 of GenBank accession no. [AY878328](http://www.ncbi.nlm.nih.gov/entrez/viewer.fcgi?db=nuccore&id=62084752).

^e^ Complement of nt 8894 to 9702 of GenBank accession no. [AY878328](http://www.ncbi.nlm.nih.gov/entrez/viewer.fcgi?db=nuccore&id=62084752).

^f^ Complement of nt 10315 to 10750 of GenBank accession no. [AY878328](http://www.ncbi.nlm.nih.gov/entrez/viewer.fcgi?db=nuccore&id=62084752).

^g^ Complement of nt 11322 to 12295 of GenBank accession no. [AY878328](http://www.ncbi.nlm.nih.gov/entrez/viewer.fcgi?db=nuccore&id=62084752).

^h^ Primers only used for sequencing (Seq.).
