# Supplementary material for: Osteopontin: an early innate immune marker of Escherichia coli mastitis harbors genetic polymorphisms with possible links with resistance to mastitis
Source: BMC Genomics. 2009 Sep 18;10:444. doi: 10.1186/1471-2164-10-444 (PMC2761946; doi:10.1186/1471-2164-10-444)
Supplement: Additional file 5 — Primers and conditions for SNP genotyping using tetra-primer ARMS-PCR in the bovine SPP1 gene. Sequence of the primers and the conditions of the PCR reactions used to genotype SPP1 using tetra-primer amplification refractory mutation system PCR (ARMS-PCR) are listed. [file 1471-2164-10-444-S5.DOC]

**Additional file 5**. Primers for SNP genotyping using tetra-primer ARMS-PCR in the bovine *SPP1* gene.

| DNA Polymorphism | Primer Sequence (5’-3’) | Final concentration (nM) | Annealing temp. (°C) | Amplicon length (bp) |
| --- | --- | --- | --- | --- |
| *SPP1c.-1301G>A* |  |  |  |  |
| Inner Forward | CCACAAAACCAGAGGGGGAAGTGTGGTAA | 100 | 61 | 244 (G) |
| Inner Reverse | ATTTTGCCACTACCCAGCCCACTGGC | 100 |  | 193 (A) |
| Outer Forward | AACCCTTTTCCCTCCCTCTACGTTTTCATGG | 10 |  | 382 |
| Outer Reverse | CTGTGATGCTGATGCAGTGCTCAGTGCT | 10 |  | 382 |
|  |  |  |  |  |
| *SPP1c.-1251C>T* |  |  |  |  |
| Inner Forward | AAATGCCCCATGACACATCTCTCCGCACT | 25 | 65 | 269 (C) |
| Inner Reverse | TGCTCCAGATGCTCTCCACCTACACCGG | 50 |  | 223 (T) |
| Outer Forward | ATGGGCCCTCTAGATGCCCTTCCAGGATG | 10 |  | 435 |
| Outer Reverse | GGCAGCGCACAGAGAGGACAGGAGTCTG | 10 |  | 435 |
|  |  |  |  |  |
| *SPP1c.-430G>A* |  |  |  |  |
| Inner Forward | AAATGCATGTTGGAAAATGGAGAAAA | 300 | 59 | 271 (G) |
| Inner Reverse | GAAGATAAAAATAAGAAAGAGAAACTGGC | 200 |  | 177 (A) |
| Outer Forward | AAAAAAACCCTTTCTGAATATTTTCACC | 75 |  | 393 |
| Outer Reverse | ACACCATTACTACCTGATATCTCCAGCT | 50 |  | 393 |
|  |  |  |  |  |
| *SPP1c.*40A>C* |  |  |  |  |
| Inner Forward | ACAATTTCTTACTTTGCTTTTAGTAAGAC | 200 | 59 | 145 (A) |
| Inner Reverse | CACCCTGCTTTAATGTATCCTTTCCT | 200 |  | 197 (C) |
| Outer Forward | ATCATAAGAGTGAAGAAGACAAACACT | 50 |  | 287 |
| Outer Reverse | AAACTCTGAAGCTTTTAGTTTAGAAGGG | 50 |  | 287 |

^a^ Nucleotide underlined corresponds to a mismatch introduced according to the tetra-primer ARMS-PCR assay to increase annealing specificity to perfect nucleotide match.
